# Differential Impact of Inhibitory G-Protein Signaling Pathways in Ventral Tegmental Area Dopamine Neurons on Behavioral Sensitivity to Cocaine and Morphine

**DOI:** 10.1523/ENEURO.0081-21.2021

**Published:** 2021-03-25

**Authors:** Margot C. DeBaker, Ezequiel Marron Fernandez de Velasco, Nora M. McCall, Anna M. Lee, Kevin Wickman

**Affiliations:** 1 Graduate Program in Neuroscience, University of Minnesota, Minneapolis, MN 55455; 2 Department of Pharmacology, University of Minnesota, Minneapolis, MN 55455

**Keywords:** cocaine, CRISPR, D2 dopamine receptor, GABA_B_ receptor, morphine, sex differences

## Abstract

Drugs of abuse engage overlapping but distinct molecular and cellular mechanisms to enhance dopamine (DA) signaling in the mesocorticolimbic circuitry. DA neurons of the ventral tegmental area (VTA) are key substrates of drugs of abuse and have been implicated in addiction-related behaviors. Enhanced VTA DA neurotransmission evoked by drugs of abuse can engage inhibitory G-protein-dependent feedback pathways, mediated by GABA_B_ receptors (GABA_B_Rs) and D_2_ DA receptors (D_2_Rs). Chemogenetic inhibition of VTA DA neurons potently suppressed baseline motor activity, as well as the motor-stimulatory effect of cocaine and morphine, confirming the critical influence of VTA DA neurons and inhibitory G-protein signaling in these neurons on this addiction-related behavior. To resolve the relative influence of GABA_B_R-dependent and D_2_R-dependent signaling pathways in VTA DA neurons on behavioral sensitivity to drugs of abuse, we developed a neuron-specific viral CRISPR/Cas9 approach to ablate D_2_R and GABA_B_R in VTA DA neurons. Ablation of GABA_B_R or D_2_R did not impact baseline physiological properties or excitability of VTA DA neurons, but it did preclude the direct somatodendritic inhibitory influence of GABA_B_R or D_2_R activation. D_2_R ablation potentiated the motor-stimulatory effect of cocaine in male and female mice, whereas GABA_B_R ablation selectively potentiated cocaine-induced activity in male subjects only. Neither D_2_R nor GABA_B_R ablation impacted morphine-induced motor activity. Collectively, our data show that cocaine and morphine differ in the extent to which they engage inhibitory G-protein-dependent feedback pathways in VTA DA neurons and highlight key sex differences that may impact susceptibility to various facets of addiction.

## Significance Statement

Although inhibitory G-protein-dependent signaling involving the GABA_B_ receptor (GABA_B_R) and D_2_ dopamine (DA) receptor (D_2_R) in ventral tegmental area (VTA) DA neurons is thought to limit DA neurotransmission evoked by drugs of abuse, their relative impact on behavioral sensitivity to such drugs is unclear. Using a neuron-specific viral CRISPR/Cas9 approach, we show that loss of D_2_R in VTA DA neurons enhances behavioral sensitivity to systemic administration of cocaine in male and female mice, whereas loss of GABA_B_R enhances cocaine sensitivity only in males. Neither GABA_B_R nor D_2_R ablation impacted behavioral sensitivity to morphine. Thus, differential engagement of inhibitory feedback pathways in VTA DA neurons likely contributes to drug-specific neurophysiological and behavioral effects and may underlie sex differences associated with some facets of addiction.

## Introduction

Dopamine (DA) neurons of the ventral tegmental area (VTA) are an integral part of the mesocorticolimbic system, a network of brain regions that mediates responses to natural rewards and drugs of abuse (Lammel et al., 2014; Volkow and Morales, 2015; Juarez and Han, 2016; Lüscher, 2016; Schultz, 2016). Drugs of abuse enhance DA neurotransmission in the mesocorticolimbic system via actions on distinct molecular targets (Di Chiara and Imperato, 1988; Lüscher and Ungless, 2006). Cocaine, for example, inhibits monoamine transporters, including the DA transporter (DAT), which recycles DA from the extracellular space, allowing DA levels to rise and persist in the VTA and its projection targets (Groves et al., 1975; Beart et al., 1979; Di Chiara and Imperato, 1988; Chen et al., 1996; Iravani et al., 1996; Adell and Artigas, 2004; Kita et al., 2009; Adrover et al., 2014). In contrast, morphine suppresses inhibitory GABAergic input, enhancing VTA DA neuron activity via disinhibition (Johnson and North, 1992; Jhou et al., 2009; Jalabert et al., 2011).

While enhanced DA neurotransmission underlies addiction-related behaviors (Hyman et al., 2006), it can also trigger negative feedback mediated by inhibitory G-protein signaling pathways (Steketee and Kalivas, 1990, 1991; Narayanan et al., 1996; Rahman and McBride, 2000). For example, cocaine elevates DA levels in the VTA and terminal regions, provoking feedback inhibition of VTA DA neurons via activation of D_2_ DA receptors (D_2_Rs) in the somatodendritic compartment and axon terminals (Einhorn et al., 1988; Bradberry and Roth, 1989; Brodie and Dunwiddie, 1990; Beckstead et al., 2004; Ford, 2014). Cocaine also provokes GABAergic feedback to VTA DA neurons indirectly, by stimulating D_1_R-expressing GABAergic medium spiny neurons in the nucleus accumbens (NAc) that project to the VTA (Rahman and McBride, 2001; Watabe-Uchida et al., 2012; Menegas et al., 2015; Edwards et al., 2017; Pignatelli and Bonci, 2018). This “long-loop” GABAergic feedback to VTA DA neurons is mediated primarily by activation of somatodendritic GABA_B_ receptors (GABA_B_Rs) on VTA DA neurons (Edwards et al., 2017).

Pharmacological and genetic approaches have implicated GABA_B_R and D_2_R-dependent signaling in the VTA in drug-induced behaviors. For example, pharmacological activation of D_2_R in the VTA suppressed cocaine-induced activity (Koulchitsky et al., 2016) and cocaine-reinstated drug seeking behavior (Xue et al., 2011), while pharmacological inhibition of D_2_R in the VTA increased psychostimulant-induced locomotor activity (Chen and Reith, 1994; Tanabe et al., 2004). Similarly, intra-VTA infusion of the GABA_B_R agonist baclofen blocked the locomotor-stimulatory effect of psychostimulants and opioids (Kalivas et al., 1990; Steketee and Kalivas, 1991; Chen and Reith, 1994; Leite-Morris et al., 2002, 2004) and attenuated self-administration of cocaine, opioids, and other drugs of abuse (Xi and Stein, 1999; Brebner et al., 2000; Leite-Morris et al., 2004; Backes and Hemby, 2008). RNAi-mediated suppression of D_2_R in the rat VTA (cell non-selective) enhanced cocaine-related behavior (de Jong et al., 2015; Chen et al., 2018), enhanced choice impulsivity (Bernosky-Smith et al., 2018), and increased functional brain activity (Martin et al., 2020). Ablation of D_2_R in DA neurons throughout the mouse brain correlated with enhanced cocaine-induced activity (Bello et al., 2011), as well as acquisition of cocaine self-administration and reactivity to drug-paired cues (Holroyd et al., 2015). Interestingly, partial suppression of GABA_B_R in VTA DA neurons unmasked cocaine-induced activity normally absent in BALB/c mice, but did not impact morphine-induced activity (Edwards et al., 2017).

Collectively, available data suggests that D_2_R and GABA_B_R-dependent signaling pathways in VTA DA neurons may exert a differential influence on behavioral sensitivity to drugs of abuse. Published studies investigating D_2_R and GABA_B_R signaling in VTA DA neurons, however, have used approaches that provide either anatomic or cellular specificity, or do not completely suppress inhibitory signaling, or focus only on one signaling pathway or drug of abuse. Accordingly, the goal of this study was to compare the impact of D_2_R or GABA_B_R ablation in VTA DA neurons on behavioral sensitivity to cocaine and morphine. To this end, we developed a neuron-specific viral CRISPR/Cas9 approach to ablate D_2_R and GABA_B_R selectively in VTA DA neurons of adult mice. Our findings show that D_2_R and GABA_B_R-dependent signaling exert drug-specific and sex-specific influences on motor activity in mice.

## Materials and Methods

### Animals

All studies were approved by the Institutional Animal Care and Use Committee at the University of Minnesota. The B6.SJL*-Slc6a3^tm1.1(cre)Bkmn^*/J (stock #006660, The Jackson Laboratory) knock-in line was used in this study; heterozygous subjects, referred to throughout as DATCre(+) mice, were generated by crossing with C57BL/6J subjects. DATCre(+) mice were also crossed in multiple rounds with a Cre-dependent Cas9GFP knock-in line (B6;129-Gt(ROSA)26Sor^tm1(CAG-cas9^*^,-EGFP)Fezh^/J, The Jackson Laboratory, stock #026179), to generate DATCre(+) subjects homozygous for the Cas9GFP(+) mutation; these mice are referred to throughout as DATCre(+):Cas9GFP(+) mice. All mice used in experiments were bred in-house. Mice were group housed, maintained on a 14/10 h light/dark cycle and were provided *ad libitum* access to food and water.

### Reagents

Baclofen, quinpirole, and sulpiride were purchased from Sigma, and CGP54626 and clozapine-N-oxide (CNO) were purchased from Tocris. Cocaine and morphine were obtained through Boynton Health Pharmacy at the University of Minnesota. All adeno-associated viruses (AAVs) were packaged in AAV8 serotype by the University of Minnesota Viral Vector and Cloning Core (Minneapolis, MN) following standard packaging procedures (Chen et al., 2019), titers were between 0.2 and 4 × 10^14^ genocopies/ml. The packaging plasmids (pRC8 and pHelper) were obtained from the University of Pennsylvania Vector Core. The plasmids pAAV-hSyn-hM4Di(mCherry) and pAAV-hSyn-DIO-mCherry (Addgene plasmids #50475 and #50459, respectively) were gifts from Bryan Roth. To obtain the pAAV-hSyn-DIO-hM4Di(mCherry) plasmid, the mCherry from the pAAV-hSyn-DIO-mCherry was replaced by an hM4Di(mCherry) cassette via subcloning. For the CRISPR/Cas9 experiments the pAAV-U6-gRNA-hSyn-NLSmCherry was generated using the backbone of the plasmid pAAV-U6-gRNA-hSyn-Cre-2A-EGFP-KASH (Platt et al., 2014; Addgene plasmid #60231) that was a gift from Feng Zhang. The Genome Engineering and iPSC Center of Washington University designed and tested guide RNA (gRNA) sequences targeting the Drd2 (D_2_R) and Gabbr1 (GABA_B_R1) genes. Sequences used were as follows: D_2_R, CATGACAGTAACTCGGCGCT; GABA_B_R1, ACGGCGTGCAGTATACATCG; LacZ, TGCGAATACGCCCACGCGAT.

### Intracranial manipulations

Mice (>45 d) were placed in a stereotaxic frame (David Kopf Instruments) under isoflurane anesthesia. Microinjectors, made by affixing a 33-gauge stainless steel hypodermic tube within a shorter 26-gauge stainless steel hypodermic tube, were attached to polyethylene-20 tubing affixed to 10-μl Hamilton syringes, and were lowered through burr holes in the skull to the VTA (from bregma: −2.75 mm A/P, ±0.55–0.7 mm M/L, −5 mm D/V); 300–500 nl of virus was injected per side at a rate of 100 nl/min. The optimized coordinates and viral load ensured full coverage of the VTA along anterior/posterior and rostral-caudal axes, with minimal spread into the adjacent substantia nigra pars compacta. Microinjectors were left in place for 10 min following infusion to reduce solution backflow along the infusion track. Slice electrophysiology and behavioral experiments were performed three to four and five to six weeks after viral infusion for chemogenetic and CRISPR experiments, respectively.

### Slice electrophysiology

Electrophysiological properties of VTA DA neurons were evaluated in behaviorally naive adult mice (66–93 d). Horizontal slices (225 μm) containing the VTA were prepared in ice-cold sucrose substituted ACSF, and allowed to recover at room temperature in ASCF containing the following: 125 mm NaCl, 2.5 mm KCl, 1.25 mm NaH_2_PO_4_, 25 mm NaHCO_3_, 11 mm glucose, 1 mm MgCl_2_, and 2 mm CaCl_2_, pH 7.4, for at least 1 h, as described (McCall et al., 2017). Neurons in the lateral VTA exhibiting appropriate fluorescence were targeted for analysis as this sub-region of the VTA receives prominent input from the NAc that mediates GABA_B_R-dependent feedback (Edwards et al., 2017). Whole-cell data were acquired using a Multiclamp 700A amplifier and pCLAMPv.9.2 software (Molecular Devices, LLC), using recording conditions described in previous publications (McCall et al., 2017). Input/membrane resistance (R_M_) and apparent capacitance (C_M_) were determined using a 5 mV/10 ms voltage step, with current responses filtered at 5 Hz. Immediately after establishing whole-cell access, I_h_ conductance was measured using a 200-ms voltage step to −120 mV; the difference in current from beginning to end of the −120-mV step was taken as I_h_ amplitude. Subsequently, spontaneous activity was measured in current-clamp mode (I = 0) for 1 min. Neurons exhibiting no spontaneous activity were not evaluated. Action potential half-width (AP_50_) was determined by averaging five AP_50_ values. For rheobase assessments, cells were held in current-clamp mode at −80 pA to prevent spontaneous activity, and then given 1-s current pulses, beginning at −100 pA and progressing in 20-pA increments. Rheobase was defined as the minimum current step that evoked one or more action potentials. In chemogenetic experiments, the change in rheobase measured before and after bath application of CNO application was determined. Somatodendritic holding currents were measured in voltage-clamp mode (V_hold_ = −60 mV) following bath application of CNO (10 μm), baclofen (200 μm), or quinpirole (10 μm). All command potentials factored in a junction potential of −15 mV predicted using JPCalc software (Molecular Devices, LLC). Series and input resistances were tracked throughout the experiment. If series resistance was unstable or exceeded 20 MΩ, the experiment was excluded from analysis.

### Locomotor activity

Locomotor activity was assessed in open-field activity chambers housed in sound-attenuating cubicles (Med-Associates). Each cubicle was equipped with three 16-beam infrared arrays permitting automated measurements of distance traveled (Activity Monitor 5; Med-Associates). Animals were habituated to the testing room for at least 30 min before testing. Subjects were acclimated over 3 d; on day 1, animals were handled and placed in the open field for 60 min. On days 2 and 3, animals were given an intraperitoneal injection of saline and placed in the open field for 60 min. For DREADD experiments, mice were injected with CNO (2 mg/kg, i.p.) 30 min before either saline, cocaine (15 mg/kg, i.p.), or morphine (10 mg/kg, i.p.) injection on day 4. Distance traveled during the 60-min period following saline or drug injection was measured; separate cohorts of mice received saline, cocaine and morphine injections. For CRISPR/Cas9 experiments, mice were placed in the open field for 30 min before injection each day to acclimate to the chamber. Activity was measured on day 3 following saline injection, and again on day 4 following injection of cocaine (15 mg/kg, i.p.) or morphine (10 mg/kg, i.p.), separate cohorts of mice were used for cocaine and morphine studies. Thigmotaxis was quantified by dividing the distance traveled in the periphery by the total distance traveled, as described (Pravetoni and Wickman, 2008). After behavioral testing, the scope and accuracy of viral targeting was assessed by fluorescence microscopy; 225-μm horizontal slices of the midbrain were obtained using a vibratome and images were acquired on an Olympus IX-80 microscope using MetaMorph Advanced Acquisition v.7.7.7.0 software (Molecular Devices, LLC). Only data from animals with bilateral viral-driven fluorescence, where the majority of fluorescence was confined to VTA (with minimal spread to the adjacent substantia nigra), were included in the final analysis.

### Statistical analysis

Data are presented throughout as the mean ± SEM. Statistical analyses were performed using Prism v.9 software (GraphPad Software). All studies included balanced numbers of male and female mice, and data were analyzed first for sex effects. If no sex differences were observed, data from male and female subjects were pooled. If any data point fell outside the range of 2 SDs from the mean, it was excluded as an outlier. Across the entire study, this outlier detection approach led to the removal of one point from the hM4Di/morphine activity dataset and 1 point from the hM4Di control/morphine activity dataset. Differences were considered significant if *p *<* *0.05.

## Results

### G-protein-dependent inhibition of VTA DA neurons suppresses motor activity

**Figure 1.**
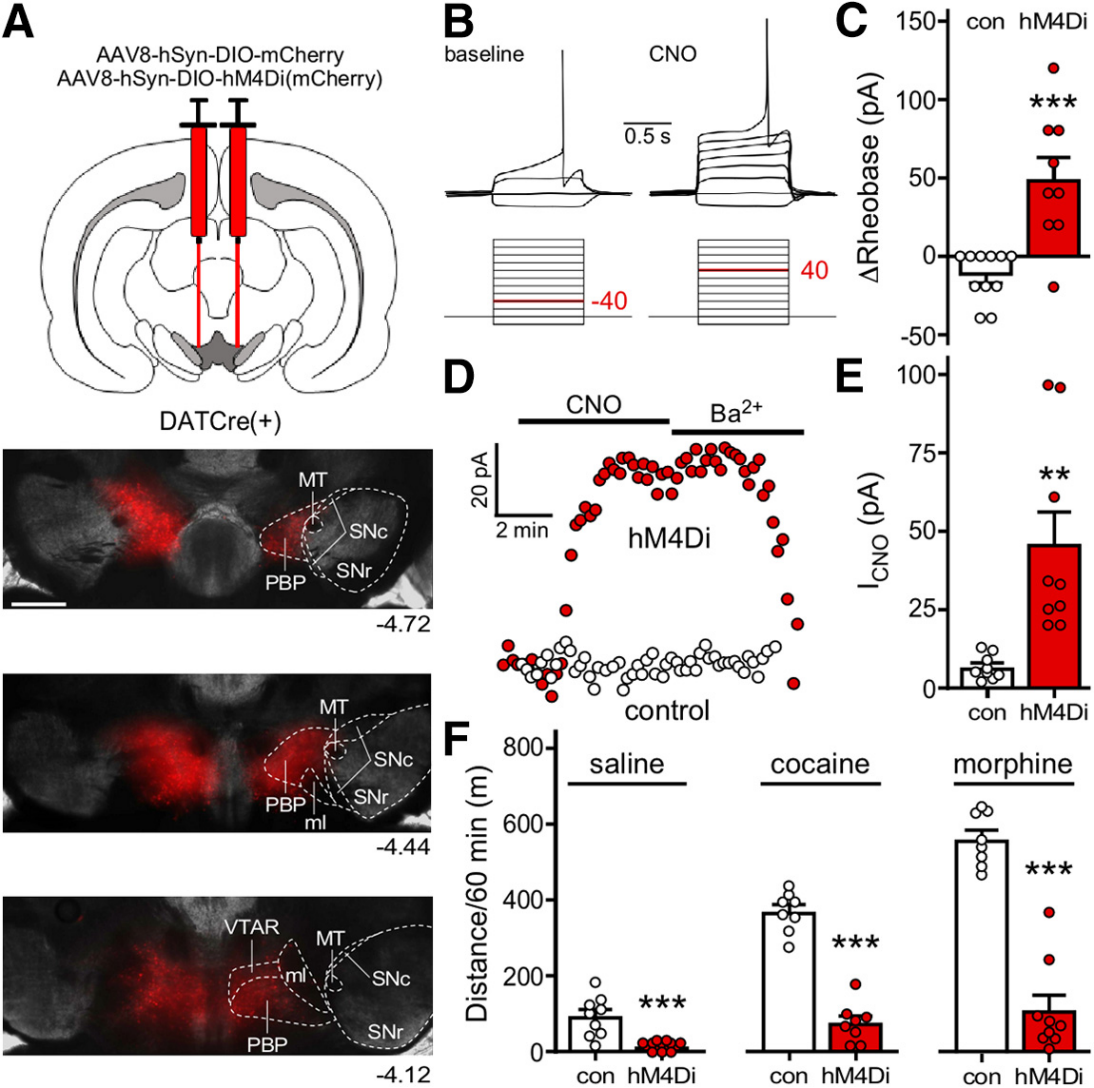
Inhibitory G-protein signaling in VTA DA neurons suppresses baseline and drug-induced motor activity. ***A***, Viral targeting in a DATCre(+) mouse treated intra-VTA AAV8-hSyn-DIO-mCherry, with rostro-caudal tiling of panels highlighting viral spread (mCherry fluorescence). ml, medial lemniscus; MT, medial terminal nucleus of the accessory optic tract; PBP, parabrachial pigmented nucleus of the VTA; SNc, substantia nigra pars compacta; SNr, substantia nigra pars reticulata; VTAR, rostral part of the VTA. Scale bar: 500 μm. ***B***, Example of rheobase measurement in a VTA DA neuron expressing hM4Di, before and after bath perfusion of CNO (10 μm). The traces shown were the first to display spiking and were recorded following injection of the current indicated in red below. ***C***, Change in rheobase induced by CNO (10 μm) in VTA DA neurons from DATCre(+) mice treated with AAV8-hSyn-DIO-hM4Di(mCherry) or AAV8-hSyn-DIO-mCherry control (*t*_(18)_ = 4.555; ***p =* *0.0002; unpaired Student’s *t* test; *n* = 9–11/group). ***D***, Somatodendritic inhibitory currents (V_hold_ = −60 mV) evoked by CNO (10 μm) in VTA DA neurons from DATCre(+) mice treated with AAV8-hSyn-DIO-hM4Di(mCherry) or AAV8-hSyn-DIO-mCherry control. ***E***, Summary of CNO-induced somatodendritic currents in VTA DA neurons from DATCre(+) treated with AAV8-hSyn-DIO-hM4Di(mCherry) or AAV8-hSyn-DIO-mCherry control (*t*_(16)_ = 3.732; **p =* *0.0018; unpaired Student’s *t* test; *n* = 9/group). ***F***, Total distance traveled in an open field during the 60-min interval following injection of saline (*t*_(16)_ = 4.385, ***p =* *0.0005), 15 mg/kg cocaine (*t*_(14)_ = 11.20, ***p <* *0.0001), and 10 mg/kg morphine (*t*_(15)_ = 9.401, ***p <* *0.0001) in DATCre(+) mice treated with intra-VTA AAV8-hSyn-DIO-hM4Di(mCherry) or AAV8-hSyn-DIO-mCherry control (*n* = 8–9 mice/group). CNO (2 mg/kg, i.p.) was administered to all subjects 30 min before saline or drug challenge.

Motor activation is an unconditioned DA-dependent response in mice to systemic administration of cocaine and morphine (van Rossum et al., 1962; Delfs et al., 1990; Kalivas and Duffy, 1993) that can be recapitulated by direct chemogenetic or optogenetic stimulation of VTA DA neurons (Kim et al., 2012; Tye et al., 2013; Boender et al., 2014; Guo et al., 2014). To test whether inhibitory G-protein-dependent signaling in VTA DA neurons can suppress the locomotor stimulatory effect of systemic cocaine and morphine, we used DATCre(+) mice and a Cre-dependent viral inhibitory chemogenetic approach. Cre-dependent AAV vectors harboring either hM4Di(mCherry) or mCherry control were infused into the VTA of adult male and female DATCre(+) mice to permit selective chemogenetic inhibition of VTA DA neurons (Fig. 1*A*).

In acutely isolated midbrain slices from virally treated DATCre(+) mice, bath application of CNO decreased the excitability (increased the rheobase) of hM4Di-expressing, but not mCherry control, DA neurons (Fig. 1*B*,*C*). CNO also evoked a somatodendritic inhibitory current (V_hold_ = −60 mV) reversed by 0.3 mm Ba^2+^ in hM4Di(mCherry)-expressing but not control VTA DA neurons (Fig. 1*D*,*E*). Thus, chemogenetic inhibition of VTA DA neurons, like the direct somatodendritic inhibition evoked by D_2_R and GABA_B_R activation, is likely mediated by activation of G-protein-gated inwardly rectifying K^+^ (GIRK/Kir3) channels (Beckstead et al., 2004; Cruz et al., 2004; Labouèbe et al., 2007; Arora et al., 2010). Consistent with a previous report (Runegaard et al., 2018), chemogenetic inhibition of VTA DA neurons suppressed motor activity evoked by injection of saline, as well as cocaine and morphine (Fig. 1*F*). These results confirm that VTA DA neurons are key regulators of motor activity in mice and show that activation of inhibitory G-protein signaling in these neurons can potently suppress baseline activity and the motor-stimulatory effects of cocaine and morphine.

### CRISPR/Cas9 ablation of D_2_R and GABA_B_R in VTA DA neurons

**Figure 2.**
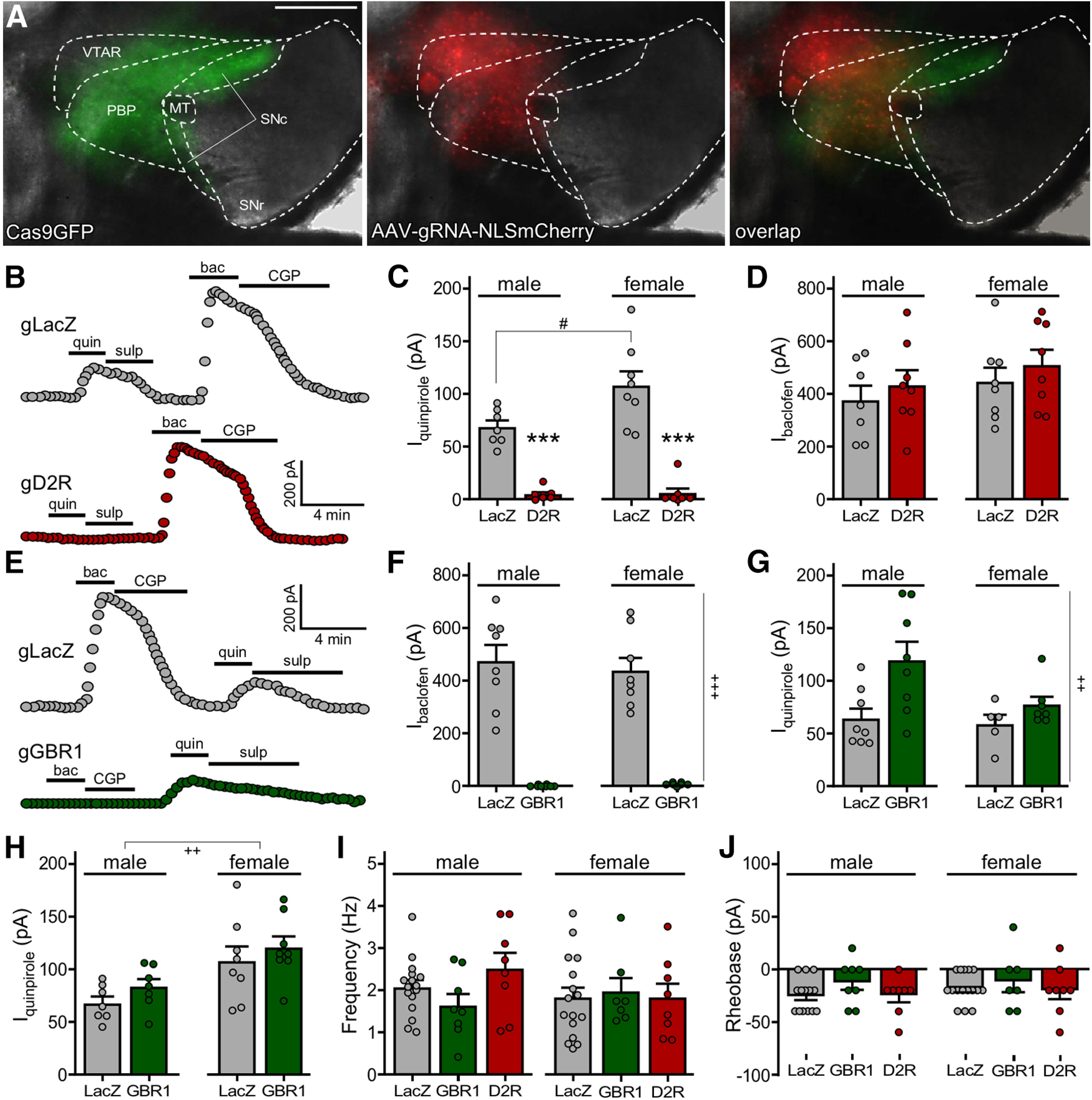
Viral CRISPR/Cas9 ablation of GABA_B_R and D_2_R in VTA DA neurons. ***A***, Viral targeting in a DATCre(+):Cas9GFP(+) mouse treated with intra-VTA AAV8-U6-gRNA-hSyn-NLSmCherry; nucleus-localized mCherry fluorescence highlights the anatomic scope of viral targeting, and GFP fluorescence denotes the Cre-dependent expression of Cas9GFP in midbrain DA neurons of the VTA and substantia nigra. MT, medial terminal nucleus of the accessory optic tract; PBP, parabrachial pigmented nucleus of the VTA; SNc, substantia nigra pars compacta; SNr, substantia nigra pars reticulata; VTAR, rostral part of the VTA. Scale bar: 500 μm. ***B***, Somatodendritic inhibitory currents (V_hold_ = −60 mV) evoked by sequential bath application of quinpirole (quin; 20 μm) and then baclofen (bac; 200 μm) in VTA DA neurons from DATCre(+):Cas9GFP(+) mice treated with AAV8-U6-gD2R-hSyn-NLSmCherry or AAV8-U6-gLacZ-hSyn-NLSmCherry control. Currents were reversed by the D_2/3_R antagonist sulpiride (sulp; 5 μm) and GABA_B_R antagonist CGP54626 (CGP; 2 μm). ***C***, Summary of currents evoked by quinpirole (applied first) in VTA DA neurons from DATCre(+):Cas9GFP(+) mice treated with AAV8-U6-gD2R-hSyn-NLSmCherry or AAV8-U6-gLacZ-hSyn-NLSmCherry control. Main effects of sex (*F*_(1,27)_ = 6.391, *p *=* *0.0176) and viral treatment (*F*_(1,27)_ = 104.6, *p *<* *0.0001) were detected, along with an interaction between sex and viral treatment (*F*_(1,27)_ = 5.590, *p *=* *0.0255); #*p *<* *0.05; ***p <* *0.001 versus LacZ (within sex). ***D***, Summary of currents evoked by baclofen, measured after quinpirole/sulpiride application (***C***), in VTA DA neurons from DATCre(+):Cas9GFP(+) mice treated with AAV8-U6-gD2R-hSyn-NLSmCherry or AAV8-U6-gLacZ-hSyn-NLSmCherry control. There was no main effect of sex (*F*_(1,27)_ = 1.741, *p *=* *0.1981) or viral treatment (*F*_(1,27)_ = 1.183, *p *=* *0.2864), nor was there an interaction between sex and viral treatment (*F*_(1,27)_ = 0.0047, *p *=* *0.9461). ***E***, Somatodendritic inhibitory currents (V_hold_ = −60 mV) evoked by sequential bath application of baclofen (bac; 200 μm) and then quinpirole (quin; 20 μm) in VTA DA neurons from DATCre(+):Cas9GFP(+) mice treated with AAV8-U6-gGBR1-hSyn-NLSmCherry or AAV8-U6-gLacZ-hSyn-NLSmCherry control. Currents were reversed by CGP54626 (CGP; 2 μm) and sulpiride (sulp; 5 μm). ***F***, Summary of currents evoked by baclofen (applied first) in VTA DA neurons from DATCre(+):Cas9GFP(+) mice treated with AAV8-U6-gGBR1-hSyn-NLSmCherry or AAV8-U6-gLacZ-hSyn-NLSmCherry control. A main effect of viral treatment was observed (*F*_(1,27)_ = 120.9, *p *<* *0.0001), but there was no main effect of sex (*F*_(1,27)_ = 0.1910, *p *=* *0.6655) or interaction between sex and viral treatment (*F*_(1,27)_ = 0.2216, *p *=* *0.6416); +++*p *<* *0.001 (main effect of viral treatment). ***G***, Summary of currents evoked by quinpirole, measured after baclofen/CGP54626 application (***F***) in VTA DA neurons from DATCre(+):Cas9GFP(+) mice treated with AAV8-U6-gGBR1-hSyn-NLSmCherry or AAV8-U6-gLacZ-hSyn-NLSmCherry control. A main effect of viral treatment was observed (*F*_(1,24)_ = 8.182, *p *=* *0.0086), but there was no main effect of sex (*F*_(1,24)_ = 3.429, *p *=* *0.0764) or interaction between sex and viral treatment (*F*_(1,24)_ = 1.970, *p *=* *0.1733); ++*p *<* *0.001 (main effect of viral treatment). ***H***, Summary of currents evoked by quinpirole in VTA DA neurons from a separate cohort of DATCre(+):Cas9GFP(+) mice treated with AAV8-U6-gGBR1-hSyn-NLSmCherry or AAV8-U6-gLacZ-hSyn-NLSmCherry control. A main effect of sex was observed (*F*_(1,26)_ = 13.41, *p *=* *0.0011), but there was no main effect of viral treatment (*F*_(1,26)_ = 1.766, *p *=* *0.1954) or interaction between sex and viral treatment (*F*_(1,26)_ = 0.0129, *p *=* *0.9105); ++*p *<* *0.01 (main effect of sex). ***I***, Impact of D_2_R or GABA_B_R ablation on spontaneous activity in VTA DA neurons. No main effect of sex (*F*_(1,56)_ = 0.7079, *p *=* *0.4037) or viral treatment (*F*_(2,56)_ = 0.6571, *p *=* *0.5223) was detected, nor was there an interaction between sex and viral treatment (*F*_(2,56)_ = 1.276, *p *=* *0.2870). ***J***, Impact of D_2_R or GABA_B_R ablation on rheobase in VTA DA neurons. No main effect of sex (*F*_(1,56)_ = 0.8080, *p *=* *0.3725) or viral treatment (*F*_(2,56)_ = 1.495, *p *=* *0.2332) was detected, nor was there an interaction between sex and viral treatment (*F*_(2,56)_ = 0.1573, *p *=* *0.8548).

To assess the impact of D_2_R and GABA_B_R-dependent signaling pathways in VTA DA neurons on behavioral sensitivity to cocaine and morphine, we developed a DA neuron-specific, viral CRISPR/Cas9 ablation approach. DATCre(+) mice were crossed with a Cre-dependent Cas9GFP line to generate DATCre(+):Cas9GFP(+) mice. AAV vectors harboring gRNAs targeting LacZ (control), D_2_R, or GABA_B_R1 were generated and infused into the VTA of male and female DATCre(+):Cas9GFP(+) mice (Fig. 2*A*).

To assess the efficacy and selectivity of the viral vectors, somatodendritic currents evoked by bath application of the D_2/3_R agonist quinpirole and the GABA_B_R agonist baclofen were measured in VTA DA neurons five to six weeks after viral infusion. In slices from LacZ gRNA-treated subjects, somatodendritic outward inhibitory currents were reliably evoked by quinpirole and baclofen (Fig. 2*B*), consistent with previous studies (Beckstead et al., 2004; Cruz et al., 2004; Labouèbe et al., 2007; Arora et al., 2010; McCall et al., 2017). In VTA DA neurons from mice treated with D_2_R gRNA, quinpirole-induced currents were completely absent (Fig. 2*B*,*C*), while baclofen (applied after quinpirole) evoked normal responses (Fig. 2*B*,*D*). Interestingly, a sex difference in the amplitude of quinpirole-induced responses was observed, with currents in VTA DA neurons from female subjects larger than those in males (Fig. 2*C*). No sex differences were observed in the amplitude of baclofen-induced currents.

In VTA DA neurons from mice treated with GABA_B_R1 gRNA, somatodendritic responses to baclofen were completely absent (Fig. 2*E*,*F*). Responses to quinpirole (applied after baclofen application) were larger than those seen in VTA DA neurons from LacZ-treated controls (Fig. 2*G*). As this difference may reflect the impact of prior activation by GABA_B_R of a shared effector in control cells, we conducted a separate study where only quinpirole was applied to the slice. In these experiments, quinpirole-induced somatodendritic currents were not significantly different between GABA_B_R-lacking and control VTA DA neurons (Fig. 2*H*).

We also examined the impact of D_2_R and GABA_B_R ablation on VTA DA neuron excitability, as measured by spontaneous activity and rheobase. No sex differences were observed with respect to either excitability measure and, as such, data from male and female subjects were pooled to increase the power of the study. Ablation of D_2_R or GABA_B_R did not impact spontaneous activity of VTA DA neurons (Fig. 2*I*) or rheobase (Fig. 2*J*). Similarly, no impact of D_2_R or GABA_B_R ablation was detected on I_h_ current amplitude, cell capacitance, action potential half-width, or input resistance (Table 1). Collectively, these data show that we can selectively ablate GABA_B_R-dependent or D_2_R-dependent signaling in VTA DA neurons, and that loss of these signaling pathways does not impact baseline electrophysiological characteristics of VTA DA neurons

**Table 1:** Electrophysiological properties of VTA DA neurons

Sex	gRNA	N/n	C_M_ (pF)	R_M_ (MΩ)	I_h_ (pA)	AP_50_ (ms)
Male	LacZ	8/15	60 ± 2	200 ± 10	313 ± 49	0.83 ± 0.04
	GABA_B_R1	3/8	56 ± 3	194 ± 11	240 ± 66	0.92 ± 0.07
	D_2_R	3/8	62 ± 4	207 ± 34	246 ± 45	0.92 ± 0.04
			*F*_(2,28)_ = 0.8299	*F*_(2,28)_ = 0.1024	*F*_(2,28)_ = 0.6179	*F*_(2,28)_ = 1.154
			*p *=* *0.4465	*p *=* *0.9030	*p *=* *0.5463	*p *=* *0.3301
Female	LacZ	9/16	62 ± 2	186 ± 11	274 ± 39	0.85 ± 0.04
	GABA_B_R1	3/7	60 ± 4	203 ± 21	225 ± 70	0.89 ± 0.06
	D_2_R	4/8	62 ± 3	174 ± 22	338 ± 71	0.99 ± 0.05
			*F*_(2,28)_ = 0.2026	*F*_(2,28)_ = 0.6060	*F*_(2,28)_ = 0.7869	*F*_(2,28)_ = 2.431
			*p *=* *0.8178	*p *=* *0.5525	*p *=* *0.4651	*p *=* *0.1063

Data extracted from whole-cell recordings of VTA DA neurons from male and female DATCre(+):Cas9GFP(+) mice treated with intra-VTA control (LacZ), D2R, or GABA_B_R1 gRNA vectors. *N*/*n*, number of mice and individual experiments; C_M_, apparent capacitance; R_M_, input/membrane resistance; I_h_, hyperpolarization-activated current; AP_50_, action-potential half-width.

### Impact of D_2_R and GABA_B_R in VTA DA neurons on baseline and cocaine-induced activity

We next examined the impact of D_2_R or GABA_B_R ablation on open-field motor activity measured after injection of saline or cocaine. Given the observed sex difference in the strength of D_2_R-dependent signaling in VTA DA neurons, and our prior report of a sex difference in cocaine-induced motor activity in mice (McCall et al., 2017), we analyzed data from male and female subjects separately. In support of this approach, we found that while male and female LacZ control mice showed no difference in total distance traveled after cocaine injection, the temporal profile of cocaine-induced activity was notably different for male and female subjects (Extended Data Fig. 3-1*A*), female subjects showed sharper time-to-peak and decay phases relative to male subjects.

**Figure 3.**
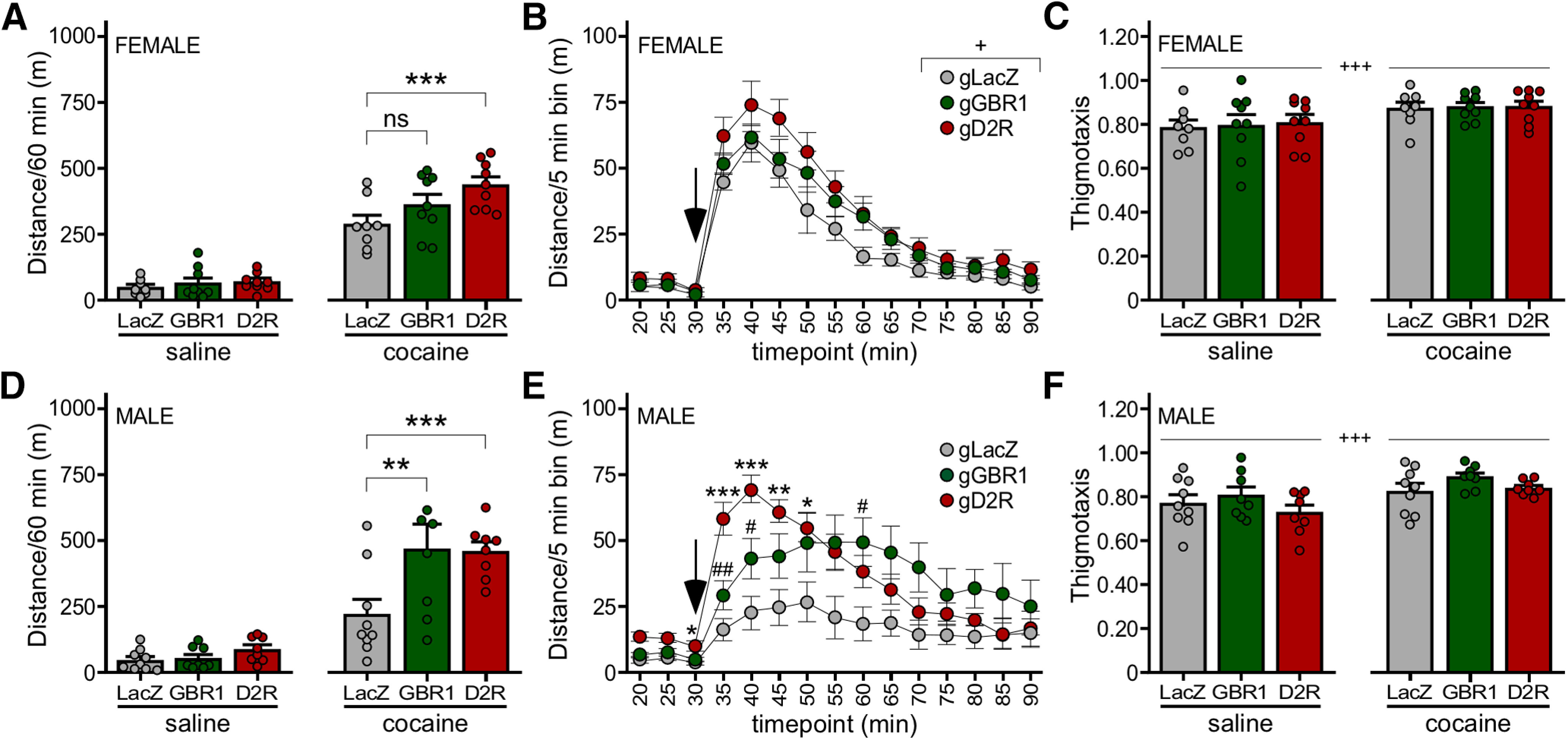
Impact of GABA_B_R and D_2_R ablation on the motor-stimulatory effect of cocaine. ***A***, Total distance traveled during the 60-min period after injection of saline (left) or cocaine (15 mg/kg, i.p., right) in female DATCre(+):Cas9GFP(+) mice treated with AAV8-U6-gGBR1-hSyn-NLSmCherry (*n* = 9), AAV8-U6-gD2R-hSyn-NLSmCherry (*n* = 9), or AAV8-U6-gLacZ-hSyn-NLSmCherry control (*n* = 8). Two-way repeated measures ANOVA revealed main effects of drug (*F*_(1,23)_ = 328.2, *p *<* *0.0001) and viral treatment (*F*_(2,23)_ = 3.778, *p *=* *0.0381), and an interaction between drug and viral treatment (*F*_(2,23)_ = 4.600, *p *=* *0.0209); ***p <* *0.001; ns, not significant (*p *=* *0.1209). ***B***, Distance traveled for female subjects before and after cocaine injection (denoted by arrow), evaluated in 5-min bins. Two-way repeated measures ANOVA revealed a main effect of bin (*F*_(3.055,70.26)_ = 82.89, *p *<* *0.0001; Geisser–Greenhouse correction for sphericity) and viral treatment (*F*_(2,23)_ = 4.613, *p *=* *0.0207), but there was no interaction between bin and viral treatment (*F*_(24,276)_ = 0.9807, *p *=* *0.4919); +*p *<* *0.05 (main effect of viral treatment). ***C***, Thigmotaxis index for female subjects following saline or cocaine injection. Two-way repeated measures ANOVA revealed a main effect of drug (*F*_(1,23)_ = 16.40, *p *=* *0.0005) but not viral treatment (*F*_(2,23)_ = 0.07,351, *p *=* *0.9293), and there was no interaction between drug and viral treatment (*F*_(2,23)_ = 0.03955, *p *=* *0.9613); +++*p *<* *0.001 (main effect of drug). ***D***, Total distance traveled during the 60-min period after injection of saline (left) or cocaine (15 mg/kg, i.p., right) in male DATCre(+):Cas9GFP(+) mice treated with AAV8-U6-gGBR1-hSyn-NLSmCherry (*n* = 8), AAV8-U6-gD2R-hSyn-NLSmCherry (*n* = 8), or AAV8-U6-gLacZ-hSyn-NLSmCherry control (*n* = 9). Two-way repeated measures ANOVA revealed main effects of drug (*F*_(1,22)_ = 91.27, *p *=* *0.0157) and viral treatment (*F*_(2,22)_ = 4.173, *p *=* *0.0291), and an interaction between drug and viral treatment (*F*_(2,22)_ = 5.050, *p *=* *0.0157); **p <* *0.01 and ***p <* *0.001. ***E***, Distance traveled for male subjects before and after cocaine injection (denoted by arrow), evaluated in 5-min bins. Two-way repeated measures ANOVA revealed a main effect of bin (*F*_(3.131,68.88)_ = 21.10, *p *<* *0.0001) and viral treatment (*F*_(2,22)_ = 4.747, *p *=* *0.0193), and an interaction between bin and viral treatment (*F*_(24,264)_ = 4.743, *p *<* *0.0001); *p <* *0.05, **p <* *0.01, ***p <* *0.001 (gD2R vs LacZ); #*p *<* *0.05 and ##*p* 0.01 (gGBR1 vs LacZ). ***F***, Thigmotaxis index for male subjects following saline or cocaine injection. Two-way repeated measures ANOVA revealed a main effect of drug (*F*_(1,22)_ = 21.75, *p *=* *0.0001), but no main effect of viral treatment (*F*_(2,22)_ = 1.534, *p *=* *0.2380) or interaction between drug and viral treatment (*F*_(2,22)_ = 0.9989, *p *=* *0.3844); +++*p *<* *0.001 (main effect of drug).

10.1523/ENEURO.0081-21.2021.f3-1 Extended Data Figure 3-1 Sex differences in the temporal profile of cocaine-induced motor activity. ***A***, Distance traveled for male and female AAV8-U6-gLacZ-hSyn-NLSmCherry control-treated subjects prior to and after cocaine injection (denoted by arrow), evaluated in 5-min bins. Two-way repeated measures ANOVA revealed no main effect of sex (*F*_(1,15)_ = 0.9539, *p* =* *0.3442), but there was a main effect of bin (*F*_(2.812,42.17)_ = 21.50, *p < *0.0001) and an interaction between bin and sex (*F*_(12,180)_ = 9.073, *p < *0.0001); *p < 0.05 and ***p < 0.001 versus male; *p < 0.05 and ***p < 0.001. ***B***, Distance traveled for male and female AAV8-U6-gD2R-hSyn-NLSmCherry-treated male and female subjects prior to and after cocaine injection (denoted by arrow), evaluated in 5-min bins. Two-way repeated measures ANOVA revealed a main effect of bin (*F*_(3.058,45.88)_ = 48.48, *p < *0.0001), but no main effect of sex (*F*_(1,15)_ = 0.3164, *p* =* *0.5821) or interaction between bin and sex (*F*_(12,180)_ = 0.6755, *p* = 0.7735). ***C***, Distance traveled for male and female AAV8-U6-gGBR1-hSyn-NLSmCherry treated subjects prior to and after cocaine injection (denoted by arrow), evaluated in 5-min bins. Two-way repeated measures ANOVA revealed no main effect of sex (*F*_(1,15)_ = 1.159, *p* =* *0.2987), but a main effect of bin (*F*_(3.059,45.88)_ = 21.96, *p < *0.0001) and an interaction between bin and sex (*F*_(12,180)_ = 6.077, *p < *0.0001). Pairwise comparisons, however, revealed no significant differences between male and female subjects at any timepoint. Download Figure 3-1, TIF file.

In females, we observed main effects of drug and viral treatment, as well as a significant interaction between drug and viral treatment, on total distance traveled in the postinjection interval. D_2_R or GABA_B_R ablation did not impact saline-induced activity in females (Fig. 3*A*, left). Loss of D_2_R, but not GABA_B_R, yielded enhanced cocaine-induced activity (Fig. 3*A*, right). Although the temporal profile of motor activity during the postinjection interval was qualitatively similar across the groups, loss of D_2_R and GABA_B_R in VTA DA neurons correlated with elevated activity levels at all time points following injection (Fig. 3*B*). Cocaine also enhanced thigmotaxis, as assessed by calculating the ratio of distance traveled in the field periphery to total distance traveled (Fig. 3*C*). There was, however, no impact of D_2_R or GABA_B_R ablation in VTA DA neurons on thigmotaxis index (Fig. 3*C*).

In males as in females, we observed main effects of drug and viral treatment, and a significant interaction between drug and viral treatment on total distance traveled. Loss of D_2_R or GABA_B_R did not impact saline-induced activity (Fig. 3*D*, left). In contrast to our observations in females, however, ablation of D_2_R or GABA_B_R in males yielded comparably enhanced cocaine-induced activity over the 60-min postinjection interval (Fig. 3*D*, right). Interestingly, loss of D_2_R in males correlated with higher levels of activity seen shortly after cocaine injection (Fig. 3*E*), yielding a temporal profile that was qualitatively similar to that observed in females (Extended Data Fig. 3-1*B*). As was the case with female subjects, thigmotaxis was significantly impacted by drug but not viral treatment (Fig. 3*C*). Thus, D_2_R-dependent signaling in VTA DA neurons tempers behavioral sensitivity to cocaine in male and female mice, whereas GABA_B_R-dependent signaling exerts an influence on behavioral sensitivity to cocaine in male mice only.

### Impact of D_2_R and GABA_B_R in VTA DA neurons on baseline and morphine-induced activity

**Figure 4.**
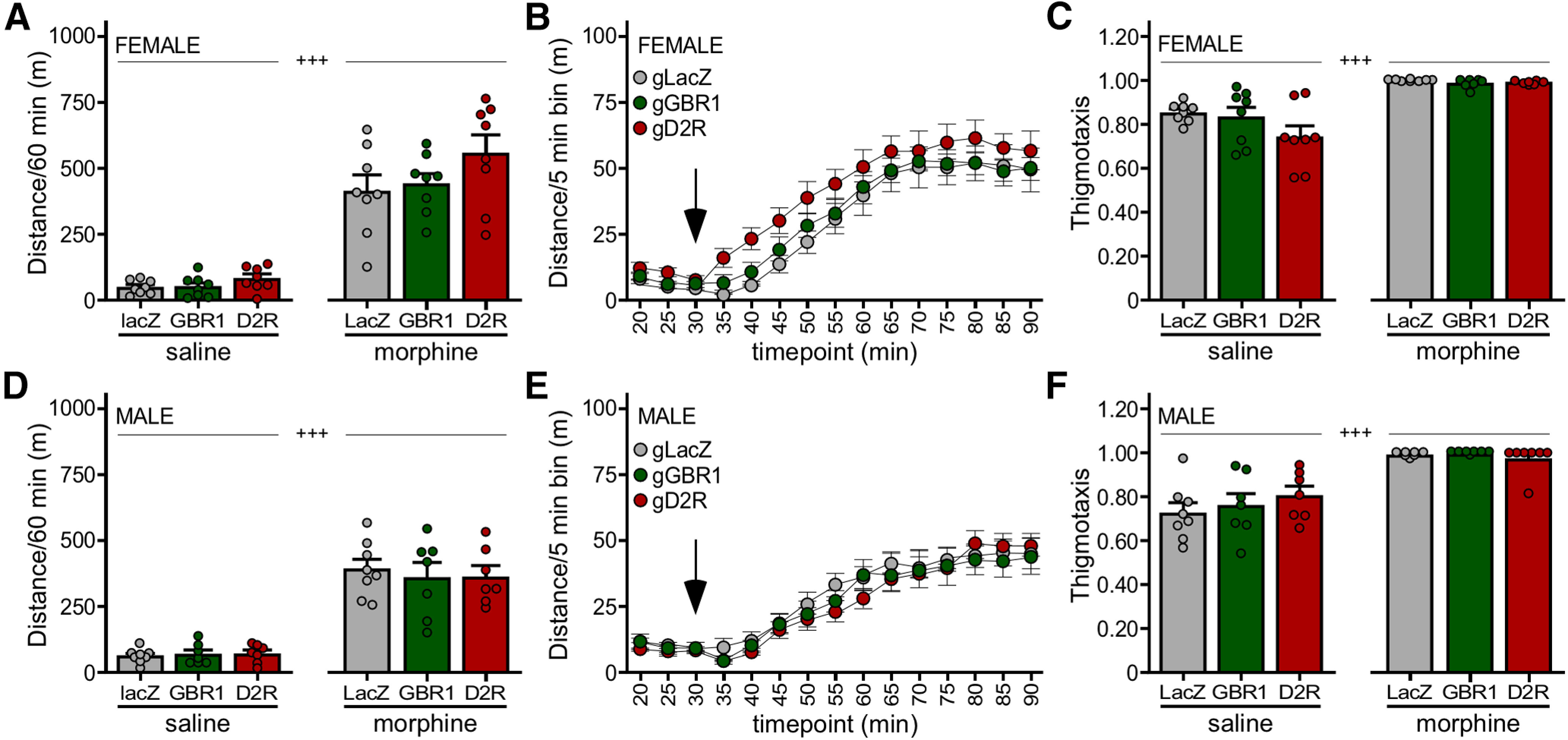
Impact of GABA_B_R and D_2_R ablation on the motor-stimulatory effect of morphine. ***A***, Total distance traveled during the 60-min period after injection of saline (left) or morphine (10 mg/kg, i.p., right) in female DATCre(+):Cas9GFP(+) mice treated with AAV8-U6-gGBR1-hSyn-NLSmCherry (*n* = 8), AAV8-U6-gD2R-hSyn-NLSmCherry (*n* = 8), or AAV8-U6-gLacZ-hSyn-NLSmCherry control (*n* = 8). Two-way repeated measures ANOVA revealed a main effect of drug (*F*_(1,21)_ = 185.3, *p < *0.0001), but no main effect of viral treatment (*F*_(2,21)_ = 2.145, *p = *0.1420) or interaction between drug and viral treatment (*F*_(2,21)_ = 1.183, *p = *0.3260); +++*p *<* *0.001 (main effect of drug). ***B***, Distance traveled for female subjects before and after morphine injection (denoted by arrow), evaluated in 5-min bins. Two-way repeated measures ANOVA revealed a main effect of bin (*F*_(3.057,67.26)_ = 97.73, *p < *0.0001), but no effect of viral treatment (*F*_(2,22)_ = 1.920, *p = *0.1704) or interaction between bin and viral treatment (*F*_(24,264)_ = 0.5380, *p = *0.9640). ***C***, Thigmotaxis index for female subjects following saline or morphine injection. Two-way repeated measures ANOVA revealed a main effect of drug (*F*_(1,21)_ = 59.23, *p *<* *0.0001), but no significant difference with viral treatment (*F*_(2,21)_ = 2.462, *p *=* *0.1095), and no interaction between drug and viral treatment (*F*_(2,21)_ = 1.995, *p *=* *0.1609); +++*p *<* *0.001 (main effect of drug). ***D***, Total distance traveled during the 60-min period after injection of saline (left) or morphine (10 mg/kg, i.p., right) in male DATCre(+):Cas9GFP(+) mice treated with AAV8-U6-gGBR1-hSyn-NLSmCherry (*n* = 7), AAV8-U6-gD2R-hSyn-NLSmCherry (*n* = 7), or AAV8-U6-gLacZ-hSyn-NLSmCherry control (*n* = 8). Two-way repeated measures ANOVA revealed main effects of drug (*F*_(1,19)_ = 176.1, *p < *0.0001), but no main effect of viral treatment (*F*_(2,19)_ = 0.05804, *p = *0.9438) or interaction between drug and viral treatment (*F*_(2,19)_ = 0.3193, *p = *0.7305); +++*p *<* *0.001 (main effect of drug). ***E***, Distance traveled for male subjects before and after morphine injection (denoted by arrow), evaluated in 5-min bins. Two-way repeated measures ANOVA revealed a main effect of bin (*F*_(2.264,43.02)_ = 80.68, *p < *0.0001), but no main effect of viral treatment (*F*_(2,19)_ = 0.1417, *p = *0.8688) or interaction between drug and viral treatment (*F*_(24,228)_ = 0.9227, *p = *0.5711). ***F***, Thigmotaxis index for male subjects following saline or morphine injection. Two-way repeated measures ANOVA revealed a main effect of drug (*F*_(1,19)_ = 68.91, *p *<* *0.0001), but no significant difference with viral treatment (*F*_(2,19)_ = 0.3907, *p *=* *0.6819), and no interaction between drug and viral treatment (*F*_(2,19)_ = 1.195, *p *=* *0.3244); +++*p *<* *0.001 (main effect of drug).

As we previously reported that the loss of GIRK channel activity in DA neurons in mice correlated with enhance motor stimulation in response to systemic administration of 10 mg/kg, morphine (Kotecki et al., 2015), we next compared the relative contribution of D_2_R and GABA_B_R to this morphine-induced behavior. In female subjects, we observed a main effect of drug on motor activity, but no main effect of viral treatment or interaction between drug and viral treatment. Notably, no significant impact of D_2_R or GABA_B_R ablation on morphine-induced activity was observed in females (Fig. 4*A*, right). Although D_2_R ablation correlated with elevated activity levels at all time points following morphine injection, activity levels were not significantly different from controls (Fig. 4*B*). Consistent with prior reports (Mickley et al., 1990), we observed a marked increase in thigmotaxis in all subjects after morphine injection, but there was no difference between viral treatment groups (Fig. 4*C*).

In males, we also observed a main effect of drug, but no main effect of viral treatment or interaction between drug and viral treatment. As was the case in females, loss of either D_2_R or GABA_B_R in males had no significant impact on morphine-induced activity during the 60-min interval (Fig. 4*D*, right) or on the temporal activity profile following morphine injection (Fig. 4*E*). We also observed a similar increase in thigmotaxis after injection of morphine as seen in females, with no difference between viral treatment groups (Fig. 4*F*). Thus, neither D_2_R nor GABA_B_R ablation in VTA DA neurons exerts a significant impact on morphine-induced motor activity in mice.

## Discussion

Here, we used a neuron-specific viral CRISPR/Cas9 approach to compare the impact of D_2_R or GABA_B_R ablation in VTA DA neurons on baseline activity and behavioral sensitivity to cocaine and morphine. Loss of GABA_B_R or D_2_R in VTA DA neurons had no significant impact on baseline activity, paralleling the lack of effect of these manipulations on excitability and other electrophysiological properties of VTA DA neurons. These behavioral findings are consistent with studies involving genetic suppression or ablation of D_2_R or GABA_B_R in the rodent VTA (de Jong et al., 2015; Edwards et al., 2017). Thus, D_2_R-dependent and GABA_B_R-dependent signaling pathways in VTA DA neurons exert minimal influence on baseline DA neuron excitability. Notably, mice lacking D_2_R in DA neurons displayed hyperlocomotion (Bello et al., 2011), indicating that D_2_R-dependent signaling in DA neuron populations outside of the VTA may regulate baseline motor activity.

D_2_R ablation in VTA DA neurons potentiated cocaine-induced activity in male and female mice, consistent with studies involving DA neuron-specific ablation of D_2_R in mice (Bello et al., 2011), and an RNAi-based approach targeting D_2_R in the rat VTA (de Jong et al., 2015). GABA_B_R ablation also potentiated cocaine-induced activity, but the influence of this signaling pathway was restricted to males. Enhanced D_2_R-dependent signaling in VTA DA neurons from female mice may compensate for the loss of GABA_B_R signaling, explaining the weak influence of GABA_B_R on cocaine-induced activity in females. Alternatively, there may be reduced GABAergic feedback to VTA DA neurons in females, rendering the loss of GABA_B_R less effective (Zachry et al., 2021).

Behavioral sensitivity to morphine was unaffected by the loss of D_2_R and/or GABA_B_R in VTA DA neurons, consistent with previous reports (Steketee and Kalivas, 1991; Maldonado et al., 1997; Edwards et al., 2017). The lack of impact of D_2_R ablation in VTA DA neurons is surprising given that opioids, like cocaine, increase VTA DA levels (Chefer et al., 2009). Cocaine and opioids differ, however, in their influence on VTA DA neurons. While cocaine hyperpolarizes VTA DA neurons in a D_2_R-dependent manner (Beckstead et al., 2004), opioids increase VTA DA neuron firing by suppressing GABAergic input from local GABA neurons and/or RMTg GABA neurons (Johnson and North, 1992; Jhou et al., 2009; Jalabert et al., 2011), as well as disinhibiting glutamatergic input to VTA DA neurons (Yang et al., 2020). Thus, any inhibitory influence of somatodendritic D_2_R activation triggered by the opioid-induced rise in VTA DA is likely offset by the excitatory influence of disinhibition.

Since morphine increases NAc DA levels (Spielewoy et al., 2000; Chefer et al., 2003; Vander Weele et al., 2014), and DA neurotransmission in the NAc drives GABA_B_R-dependent feedback to VTA DA neurons (Edwards et al., 2017), it is also surprising that GABA_B_R ablation does not impact behavioral sensitivity to morphine. This outcome might reflect differences in the amplitude of the DA increase evoked by cocaine and morphine. Indeed, NAc DA levels in freely moving rats increased more in response to intravenous cocaine than morphine (Pontieri et al., 1995). The spatiotemporal pattern of DA increases in the NAc may also differ for cocaine and morphine. Consistent with this premise, cocaine elicited a more pronounced increase in DA in the NAc shell as compared with core (Aragona et al., 2008, 2009), whereas morphine evoked similar DA increases in NAc core and shell (Vander Weele et al., 2014). Moreover, the morphine-induced increase in NAc DA was relatively transient, a phenomenon potentially linked to a simultaneous increase in NAc GABA levels evoked by morphine (Vander Weele et al., 2014).

The differential apparent engagement of GABA_B_R-dependent and D_2_R-dependent signaling pathways by cocaine and morphine may also reflect differential molecular target location. Morphine can act on opioid receptors in other brain regions to regulate motor activity, bypassing VTA DA neurons. For example, locomotor activity decreased initially following intra-NAc injection of morphine in rats (Costall et al., 1978). In addition, NAc lesions failed to eliminate morphine-induced motor activation, suggesting a potential role of other brain regions in this effect (Stevens et al., 1986). Also, intra-NAc infusion of morphine abolished the motor stimulatory effect of intra-NAc DA, showing that opioid and dopaminergic pathways in the NAc exert competing influence on locomotion (Layer et al., 1991).

The direct inhibitory influence of GABA_B_R and D_2_R activation on VTA DA neurons is mediated primarily by activation of GIRK channels (Brodie and Dunwiddie, 1990; Ackerman et al., 1993; Mercuri et al., 1997; Chen and Pan, 2000; Bunney et al., 2001; Beckstead et al., 2004; Cruz et al., 2004; Ford, 2014), though other effectors are modulated as well (Philippart and Khaliq, 2018; Su et al., 2019). Genetic ablation of *Girk2* globally or selectively in DA neurons correlated with increased motor-stimulatory effect of cocaine (Pravetoni and Wickman, 2008; Kotecki et al., 2015; McCall et al., 2017, 2019) and morphine (Kotecki et al., 2015). The contribution of GIRK channels to cocaine-induced activity was further localized to VTA DA neurons (McCall et al., 2019). Given that GIRK channels mediate the D_2_R-dependent and GABA_B_R-dependent inhibition of VTA DA neurons and that DA neuron-specific loss of GIRK channels enhances the motor-stimulatory effect of cocaine and morphine, the lack of impact of D_2_R or GABA_B_R ablation on morphine-induced activity was unexpected. It is possible that GABA_B_R-dependent and D_2_R-dependent signaling pathways are functionally redundant and that both need to be eliminated to see an influence on morphine-induced activity. GIRK channels in non-VTA DA neurons, perhaps in the adjacent substantia nigra pars compacta (Koyrakh et al., 2005), may also explain the impact of GIRK channel ablation on morphine.

Females are more susceptible to various facets of addiction (Fattore et al., 2008; Anker and Carroll, 2011; Becker and Koob, 2016), fueling interest in identifying relevant sex differences at molecular and cellular levels. Here, we found multiple sex differences related to inhibitory G-protein signaling in VTA DA neurons. Loss of GABA_B_R, for example, had minimal impact on behavioral sensitivity to cocaine in females, but significantly enhanced the motor-stimulatory effect of cocaine in males. D_2_R-dependent somatodendritic inhibitory currents were also larger in VTA DA neurons from females. This difference could reflect elevated D_2_R expression and/or function in VTA DA neurons from females and is predicted to decrease cocaine sensitivity. Indeed, females exhibit elevated D_2_R-dependent signaling at baseline as compared with males (Walker et al., 2006; Zachry et al., 2021).

Cocaine-induced activity was characterized by a more rapid and sharp peak in females as compared with males, which could be explained by a tighter regulation of synaptic DA by DAT in females compared with males (Zachry et al., 2021). D_2_R ablation in VTA DA neurons in male mice yielded a temporal profile comparable to that seen in female mice. While this suggests that D_2_R-dependent signaling in VTA DA neurons is critical in tempering the early behavioral response to cocaine, the shift in the temporal profile observed in males is somewhat counterintuitive given that somatodendritic D_2_R-dependent signaling is weaker in VTA DA neurons from males. This apparent discrepancy is perhaps explained by sex differences in the influence of presynaptic/terminal D_2_R (which should also be eliminated by the CRISPR/Cas9 ablation approach) on DA dynamics in the NAc. Presynaptic D_2_R-dependent signaling in VTA DA neurons in males may be stronger than that seen in females. Alternatively, while D_2_R-dependent somatodendritic response amplitudes are larger in VTA DA neurons from female mice, VTA DA neurons in males may be more sensitive to D_2_R-dependent inhibition.

While D_2_R-dependent and GABA_B_R-dependent signaling pathways in the VTA can suppress behavioral sensitivity to cocaine, psychostimulant exposure can weaken these inhibitory feedback pathways. For example, a transient decrease in D_2_R influence on VTA DA neuron firing has been reported after repeated cocaine treatment or self-administration (Henry et al., 1989; Ackerman and White, 1990). This adaptation, and corresponding potentiation of cocaine-induced motor activity, was reproduced by repeated quinpirole treatment (Henry et al., 1998). Cocaine self-administration in rats also increased firing rate and burst activity of midbrain DA neurons, paralleled by decreased ability of quinpirole to inhibit DA neuron firing rate (Marinelli et al., 2003). Self-administration of amphetamine in rats reduced the ability of D_2_R to suppress evoked DA release in the NAc, an effect mediated in part by an RGS2-dependent disruption of D_2/3_R/Gα_i2_ functional coupling (Calipari et al., 2014; Sun et al., 2015). Repeated cocaine in male rats also decreased GABA_B_R/G-protein coupling (Kushner and Unterwald, 2001). Finally, cocaine suppressed GABA_B_R-dependent somatodendritic signaling in putative VTA DA neurons (Arora et al., 2011), and methamphetamine self-administration suppressed D_2_R-dependent and GABA_B_R-dependent somatodendritic responses in VTA DA neurons (Sharpe et al., 2014).

Notably, D_2_R activation appears critical for many of these forms of plasticity, which likely contribute to the hyperexcitability of meso-accumbens DA pathway following psychostimulant exposure (Henry et al., 1998; Francis et al., 2019). The reciprocal relationship between psychostimulants and inhibitory G-protein-dependent feedback pathways in VTA DA neurons may be dependent on age and/or species, or methodological variables. Indeed, repeated methamphetamine injections correlated with enhanced D_2_R-dependent hyperpolarization in young (8–10 d) rats (Amano et al., 2003). In mice, repeated methamphetamine injections suppressed somatodendritic GABA_B_R-dependent (but not D_2_R-dependent) signaling in VTA DA neurons, but only when methamphetamine was given in novel environment (Munoz et al., 2016).

Our work highlights innate signaling mechanisms that regulate behavioral sensitivity to cocaine. Knowledge of the molecular players and neuron populations regulating behavioral sensitivity to cocaine and other drugs of abuse may help in identifying individuals at risk for developing addiction. Further investigation of mechanisms regulating the strength and sensitivity of inhibitory G-protein signaling pathways in VTA DA neurons may suggest opportunities for selective therapeutic interventions tailored to specific drugs of abuse.

## Synthesis

Reviewing Editor: Alexxai Kravitz, Washington University in St. Louis Decisions are customarily a result of the Reviewing Editor and the peer reviewers coming together and discussing their recommendations until a consensus is reached. When revisions are invited, a fact-based synthesis statement explaining their decision and outlining what is needed to prepare a revision will be listed below. The following reviewer(s) agreed to reveal their identity: NONE. Note: If this manuscript was transferred from JNeurosci and a decision was made to accept the manuscript without peer review, a brief statement to this effect will instead be what is listed below.

I have read the manuscript, prior reviews, and rebuttal letter and believe this revision has addressed prior concerns and is suitable for publication without further review.
