# Supplementary figures and images for: Differential Impact of Inhibitory G-Protein Signaling Pathways in Ventral Tegmental Area Dopamine Neurons on Behavioral Sensitivity to Cocaine and Morphine
Source: eNeuro. 2021 Mar 25;8(2):ENEURO.0081-21.2021. doi: 10.1523/ENEURO.0081-21.2021 (PMC8114902; doi:10.1523/ENEURO.0081-21.2021)

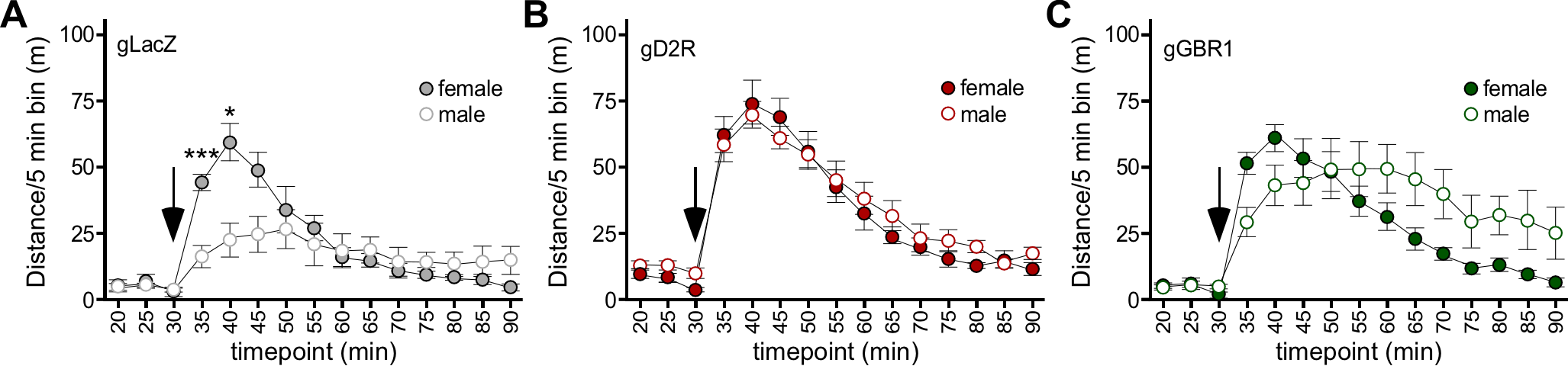

Supplement: Extended Data Figure 3-1 — Sex differences in the temporal profile of cocaine-induced motor activity. A, Distance traveled for male and female AAV8-U6-gLacZ-hSyn-NLSmCherry control-treated subjects prior to and after cocaine injection (denoted by arrow), evaluated in 5-min bins. Two-way repeated measures ANOVA revealed no main effect of sex (F(1,15) = 0.9539, p = 0.3442), but there was a main effect of bin (F(2.812,42.17) = 21.50, p < 0.0001) and an interaction between bin and sex (F(12,180) = 9.073, p < 0.0001); *p < 0.05 and ***p < 0.001 versus male; *p < 0.05 and ***p < 0.001. B, Distance traveled for male and female AAV8-U6-gD2R-hSyn-NLSmCherry-treated male and female subjects prior to and after cocaine injection (denoted by arrow), evaluated in 5-min bins. Two-way repeated measures ANOVA revealed a main effect of bin (F(3.058,45.88) = 48.48, p < 0.0001), but no main effect of sex (F(1,15) = 0.3164, p = 0.5821) or interaction between bin and sex (F(12,180) = 0.6755, p = 0.7735). C, Distance traveled for male and female AAV8-U6-gGBR1-hSyn-NLSmCherry treated subjects prior to and after cocaine injection (denoted by arrow), evaluated in 5-min bins. Two-way repeated measures ANOVA revealed no main effect of sex (F(1,15) = 1.159, p = 0.2987), but a main effect of bin (F(3.059,45.88) = 21.96, p < 0.0001) and an interaction between bin and sex (F(12,180) = 6.077, p < 0.0001). Pairwise comparisons, however, revealed no significant differences between male and female subjects at any timepoint. Download Figure 3-1, TIF file. [file enu-eN-NWR-0081-21-s01.tif]
